# An updated classification of meditation methods using principles of taxonomy and systematics

**DOI:** 10.3389/fpsyg.2022.1062535

**Published:** 2023-02-08

**Authors:** Jonathan D. Nash, Andrew B. Newberg

**Affiliations:** ^1^Retired, Unaffiliated, Chiangmai, Thailand; ^2^Department of Integrative Medicine and Nutritional Sciences, Jefferson University Hospitals, Thomas Jefferson University, Philadelphia, PA, United States

**Keywords:** meditation, taxonomy and classification, contemplative traditions, neurophysiology, neurobiology, contemplative neuroscience, cognition and affect, consciousness

## Abstract

This paper revisits the proposal for the classification of meditation methods which we introduced in our initial 2013 publication, “Toward a Universal Taxonomy and Definition of Meditation”. At that time, we advanced the thesis that meditation methods could be effectively segregated into three orthogonal categories by integrating the taxonomic principle of functional essentialism and the paradigm of Affect and Cognition; and we presented relevant research findings which supported that assertion. This iteration expands upon those theoretical and methodological elements by articulating a more comprehensive Three Tier Classification System which accounts for the full range of meditation methods; and demonstrates how recent neuroscience research continues to validate and support our thesis. This paper also introduces a novel criterion-based protocol for formulating classification systems of meditation methods, and demonstrates how this model can be used to compare and evaluate various other taxonomy proposals that have been published over the past 15 years.

## 1. Introduction

The relatively nascent discipline of contemplative neuroscience^1^ (CoNS) has been devoted to investigating the phenomenon of meditation by using neuroscientific tools, such as EEG, fMRI, PET, and SPECT. Although EEG meditation research can be traced back to the 1950s (Das and Gastaut, 1955), awareness and legitimacy of CoNS was significantly advanced in the wake of the landmark “Investigating the Mind Conference” at MIT in October, 2003 (Barinaga, 2003). Since then, we have seen hundreds of neuroscience research projects directed toward a greater understanding of the various mental and neurophysiological states achieved during meditation. It is notable, however, that the CoNS field remains devoid of a universally recognized taxonomy and classification system for the full range of contemplative meditation methods. This is a matter of concern, especially given that the philosophical disciplines of taxonomy and systematics assert that a well-conceived classification is essential to create order, provide for clear communication, and frame all theoretical considerations for any given scientific discipline (Ereshefsky, 2000).

Since the publication of our initial proposal “toward a universal taxonomy” of meditation in 2013 (Nash, Newberg, and Awasthi aka NNA) (Nash et al., 2013), several other alternative theses have been published which promulgate diverse, and seemingly incompatible, classification systems (see sections 3.3 and 8.0). This current lack of clarity and consensus is reminiscent (albeit on a much smaller scale) of the problems faced by Linnaeus in the 1700s, when biologists disagreed on the categories of classification, and how to name and assign biological entities to those categories. Fortunately, Linnaeas was able to bring order to the field of biology by introducing clear and simple rules for constructing classifications, and by devising a cogent nomenclature which greatly enhanced the ability of biologists to communicate their hypotheses and research findings (Ereshefsky, 2000).

In the paper of NNA (2013), we argued that the disciplines of the Philosophy of Taxonomy and the Science of Systematics provided essential pedagogical precepts for formulating a cogent classification of meditation methods for the field of CoNS. This iteration reaffirms our original assertion that the Aristotelian taxonomic school of functional essentialism appears to be the most suitable theoretical basis for the classification of immaterial processes such as meditation; and we discuss in detail why it is problematic and ineffective to rely solely on empirically-driven, statistical, and inductive/intuitive-type methods.

The primary objectives of this paper are:

- to present a more compelling case for CoNS researchers to give due diligence to the established taxonomic theories, principles, and standards that have been promulgated by the disciplines of Philosophy of Taxonomy and the Science of Systematics;
- to update and refine the classification system that we introduced in the original NNA 2013 paper; expand the typology to account for the full range of meditation methods by including both simple and more complex methods; and provide a more in-depth discussion of the foundational principles which informed the original thesis and have carried over to this iteration;
- to introduce a new criterion-based model for the formulation of classification systems; to demonstrate how these criteria were used to inform this proposal; and to use this model to evaluate various other “competing” taxonomy proposals;
- to offer an efficacious and straightforward three-tier classification system that could serve as a useful model for meditation research; and
- to demonstrate how recent neuroscience research continues to support and validate the original taxonomic theses advanced by the paper of NNA (2013).

## 2. A brief review of the NNA 2013 taxonomy model

Our 2013 paper proposed a two-tier classification system for relatively simple meditation methods that were singular in intent—a higher order system of three overarching orthogonal domains based on the notions of Affect, Cognition, and the absence of such (Null); and a sub-classification system based on nine defining characteristics called Taxonomic Keys. That model was predicated on three inter-dependent principles:

- The philosophy of functional essentialism, which espouses the necessity of determining the *functional essence* of whatever entity/process that one is attempting to classify.
- The assertion that the *functional essence* of a meditation method is its *intention* to engender a meditative state, aka an enhanced mental state (EMS) which is differentiated from the mundane waking state to a more “profound” state of consciousness that can include an enhanced sense of well-being, increased focus, intense affect, profound calmness or bliss, detachment, insight, or emptiness to name a few.
- The integration of this notion of *intentionality* with the aforementioned typology of Affect, Cognition, and Null, to devise a classification scheme of three orthogonal overarching domains of meditation methods.

This paper conforms with, and builds upon, these foundational principles.

## 3. Applying principles of taxonomy to the field of contemplative neuroscience

### 3.1. Taxonomic considerations

Since there are many kinds of meditation methods, with different goals and with varying techniques, researchers and authors need to be able to communicate their findings to each other and the general public in a way that is not confusing or wide-open to mis-interpretation. A cogent taxonomy provides the structure for accomplishing this. The following review is presented here in the hope that it may influence future efforts to give due consideration to these precepts.

#### 3.1.1. What is a “taxonomy” and why should we bother with it in the first place?

*A well-conceived and useful taxonomy has the power to frame all theoretical considerations of a particular field of study*. *It is natural, and in fact historical, for scientists and philosophers to desire to segregate and classify the things and processes of this world* (Ereshefsky, 2000).

One could argue that the need to organize and create order in the world extends beyond just scientists and philosophers, and could be considered a fundamental characteristic of human nature. The growth of knowledge through science depends, in part, on creating structure and order by devising useful classifications, and such endeavors date back to the ancient Chinese and Egyptian cultures 1,500–2,700 years BCE. Since Plato and Aristotle, we have seen the development of the Philosophy of Taxonomy and the scholarly pursuit of theories, principles, and methods; especially with regard to the classification of animals, plants, and minerals. There has been, and continues to be, much debate between scientists and philosophers as to which approach is most efficacious, and this has resulted in the promulgation of several well-respected theories and schools of taxonomy. Textbooks on this subject could fill an entire section of the library (for example, see Mayr, 1969; Ereshefsky, 2000; Kendig and Witteveen, 2020).

The term taxonomy, derived from the ancient Greek word *taxon*, refers to the philosophy, principles and methods which form the basis for “the systematic differentiation and categorization of things and processes of interest,” known as classification (Mayr, 1969). Herein lies a major point of emphasis of this paper: that a cogent theoretical taxonomic foundation is a necessary pre-requisite for developing an efficacious classification system.

Given that this proposal attempts to adhere to basic taxonomic principles, and relies on taxonomic jargon and nomenclature, it seemed prudent to present a brief review of the pertinent concepts and terms which informed the taxonomic arguments and theses which follow.

#### 3.1.2. Glossary of relevant taxonomic terms:

- Essentialism: a taxonomic philosophy, credited to Aristotle, which emphasizes the importance of identifying the *essence* of whatever entity/process is being classified; which are then sorted/categorized according to their *essential natures*. This theory asserts that the members of a natural group share a common essence that causes them to be members of that group (Ereshefsky, 2000).
- Functional essentialism is a specific type of essentialism; based on a teleological approach which defines the essence of a given entity/process by determining its aim/purpose. Aristotle believed that the real essence of a given entity/process is its power to achieve certain ends, as opposed to material essentialism, which holds that real essences are physical properties or characteristics (Ereshefsky, 2000; Austin, 2017).
- Monism vs. pluralism: Monists advocate for orthogonal categories, whereas pluralists allow for a number of equally acceptable categories for the entities/processes that are being classified. Aristotle was both a taxonomic monist and a metaphysical monist. He maintained that we should strive for scientific classifications that accurately represent the “single correct way the world is carved” (Ereshefsky, 2000).
- Orthogonality: a classification principle which asserts that no entity/process can be a member of more than one group—that is, the overarching domains of the classification scheme are mutually exclusive.
- Pheneticism: a classification strategy (coined by Sokal and Sneath, 1963) which segregates and groups entities/processes by their observable similarities and differences. A phenetic approach uses cluster analysis to divide entities into groups whose members share similar traits regardless of their causal connections, and none of those traits are deemed essential (Sokal and Sneath, 1963; Ereshefsky, 2000).
- Teleology/teleological: the function of an entity or process relative to its purpose—what it was designed or intended to do (Neander and Schulte, 2020).

Our 2013 proposal, as well as this current iteration, utilizes a teleological, functional essentialist philosophy and a monist orthogonal methodology, for reasons explained below.

### 3.2. Meditation considerations

As noted in NNA 2013, the term *meditation* has been a source of considerable conflation. Within common parlance, the noun *meditation* and its corresponding verb *to meditate* have become generic terms, with various meanings. For example, it is common to hear such phrases as: “I’m not sure about this, let me meditate on it tonight,” or “her essay was an inspiring meditation on the urgency of global warming,” etc. Within the contemplative, philosophical, and scientific domains, the term meditation has been variously used to refer to a particular method or technique, e.g., reporting that certain subjects were practitioners of Buddhist meditation; versus referring to the mental aspects, e.g., reporting that a particular subject was in a deep state of meditation. This ambiguity, known as “method vs. state,” has been well-recognized and discussed for many years within the CoNS literature (West, 1987; Koshikawa et al., 1996; Lutz et al., 2007; Awasthi, 2012). This explicit differentiation between “method” and “state” is an important distinction for the formulation of a cogent taxonomy (see section 5.0).

#### 3.2.1. The meditation method

Meditation methods (MMs) have been defined as “emotional and attentional regulatory training regimes developed for various ends, including the cultivation of well-being and emotional balance” (Lutz et al., 2008). In this sense, MMs provide a scheme for regulating (controlling or directing) mental faculties of attention and emotion (aka cognition and affect) for the attainment of various contemplative goals. It is for these reasons that MMs are commonly considered to be a vehicle for a particular kind of mental training.

For the purposes of this paper, the MM is defined as the prescribed set of instructions and techniques that the practitioner can choose to employ in an attempt to facilitate the attainment of a particular intended EMS (see NNA, 2013 for more detail). Since this is a proposal directed to the field of CoNS, our taxonomy focuses on those MM that have been, or will be, the subject of CoNS research.

Admittedly, it would be presumptuous for anyone to claim that there is only one “right” definition of what is (and what is not) a *bona fide* MM. With that caveat in mind, this taxonomic proposal adheres to the confines of the mental training definition above, and we have excluded consideration of those methods (that some may consider to be meditation) which have a primary focus on physical/somatic skills, e.g., various forms of Hatha Yoga, external/martial forms of Tai Chi, etc. For a more detailed discussion of the conceptual issues regarding somatic elements in the taxonomy of meditation—see section 8.1.1.

It is important to note that the 2013 paper only considered MMs that were simple, singular of purpose methods—that is, methods which were designed to engender one particular type of meditative state. This proposal has been expanded to account for complex MMs, which were designed to engender more than one type of EMS within a given meditation session. The former group has been designated as Simple Methods, and the latter as Complex Methods—see section 6.0.

#### 3.2.2. The meditative state

The 2013 paper defined the meditative state as the intended result of the successful application of a *bona fide* MM—an enhanced mental state (EMS) with defining neurophysiological and phenomenological properties/characteristics. Within the context of this typology, the term “enhanced” is used as a distinction to the mundane state of waking consciousness, and is intended to connote notions, such as “deeper,” “higher,” “intensified,” “expanded,” and “more profound.” This iteration adheres to our original thesis of three distinctive EMSs—affective, cognitive, and null (see section 5.2.2). The neurophysiological correlates of these states and their implications for taxonomy are discussed in section 7.0.

In general terms, the meditative state has been described in subjective first-person reports as a shift in consciousness from the mundane waking state to a more “profound” mental state, e.g., an enhanced sense of well-being, focus, calm, detachment, insight, affect, bliss, emptiness, etc.; and many research studies have demonstrated distinctive neurophysiological correlates of the meditative state (e.g., Travis and Pearson, 2000; Newberg and d’Aquili, 2001; Vaitl et al., 2005; Cahn and Polich, 2006; Lutz et al., 2007; Bærentsen et al., 2009; Dahl et al., 2015; Brandmeyer et al., 2019; Raffone et al., 2019; Travis, 2020). The meditative state may manifest as a fleeting, momentary state (as typically reported by novice practitioners), or may be sustained for considerable periods of time (as typically reported by advanced/highly experienced meditators).

#### 3.2.3. Meditation as a dynamic process

In our Definition Section of the 2013 paper, we introduced the notion of meditation as a dynamic process, inclusive of both method and state; depicted as five sequential and inter-connected steps: Intention to Begin, Preliminaries, engaging with the MM itself, the EMS itself, and Intention to Finish (for an in-depth discussion, see NNA, 2013 pp. 3–5). The idea that meditation can be considered an immaterial process (with, of course, important biological and neurophysiological correlates) has taxonomic implications when contrasted with classifications of physical entities such as biological organisms—an important distinction discussed in the following section.

### 3.3. Theoretical and methodological issues—challenges and obstacles

The first major attempt to classify the full range of MMs was initiated outside the field of CoNS, but had important implications for the entire scope of meditation research. Dr. Maria Ospina and her group of researchers prepared a comprehensive report on meditation and health for the U.S. Department of Health and Human Services (Ospina et al., 2007). They reviewed over 1,000 meditation publications, and conducted an exhaustive survey of the nuances of many meditation techniques, the various cognitive strategies employed, and the variety of subjective self-reports of the meditation experience itself. They employed a combination of inductive reasoning, intuition, consensus-building, and statistical analyses (here dubbed an “empirical/intuitive approach”), to infer five overarching classification categories: Mantra, Mindfulness, Tai Chi, Qigong, and Yoga. However, they stipulated that these were just “general categories” (not orthogonal domains), and offered the following equivocations: (1) “Meditation is an umbrella term that encompasses a family of practices that share some distinctive features, but that vary in important ways in their purpose and practice.” (2) “This lack of specificity of the concept of meditation precludes developing an exhaustive taxonomy of meditation practices.” (3) It is “impossible to select components that might be considered universal or supplemental across practices,” due to “the theoretical and terminological heterogeneity among practices” (pp v.—3). In other words, they concluded that, for various reasons, it was not possible to create a classification system of MMs that was capable of orthogonal distinctions. In 2013, we speculated that the source of their well-documented difficulties might be attributed, at least in part, to the taxonomic principles and methods that were employed (or not), rather than just the complexity and “heterogeneity” of MMs, and we suspected that there might be confounding issues with their research methodology that contributed to their difficulties.

In an attempt to find answers to this quandary, we conducted a literature review within the disciplines of the Philosophy of Taxonomy and the Science of Systematics. It became apparent that, although the Ospina team had not made any reference to fundamental taxonomic principles (and none were cited), they had inadvertently employed an approach which was quite similar to “material essentialism,” “taxonomic pluralism,” “pheneticism,” and “cluster analysis.” Although these taxonomic philosophies/methods have proved quite successful in creating useful classifications for living organisms (e.g., the phylum, clades, classes, and species of the Animal Kingdom), it called to question whether they were suitable for the classification of an immaterial phenomenon such as meditation.

In the 2013 paper, we argued that attempting to classify MMs by using the aforementioned phenetic-like (an inadvertent application of pheneticism), empirical/intuitive approach (while bypassing consideration of fundamental taxonomic principles) would likely prove problematic. It seems that this assertion has mostly fallen on deaf ears. Virtually all of the meditation taxonomies published since the 2013 paper replicated the approach of Ospina et al. (2007); and made no mention whatsoever of taxonomic philosophy, principles, or methods. It is unsurprising therefore, that these efforts experienced similar problems and limitations as well.

Here are some notable examples from other taxonomy proposals that attempted to formulate orthogonal categories for MMs, but did not succeed for varying reasons (the bold type has been added by us for emphasis):

- Fox et al. (2014) concluded that they “**could not classify meditation as being exclusively of any one class** because meditation practice demonstrates varying emphases on concentration, mindfulness, or guidance”; and “virtually all practices involve a combination of these strategies”; and “despite the potential value of various classification schemes, comparative analyses based on meditation type were not undertaken here.” (p.53).
- Matko et al. (2018, 2021), Matko and Sedlmeier (2019), in referring to the statistical analysis they employed to formulate their overarching domains, qualified their results by stating that they could only derive “a dimensional output with **potentially** meaningful clusters, which are **open to interpretation** by the researcher.” (p.7)
- Sparby and Sacchet (2022) attempted to differentiate MMs on the basis of the “activities” performed during meditation and their “effects,” but they discovered that this was not an orthogonal enterprise. They offered the following disclaimer: “some meditative activities may be performed occasionally…while others may be performed all the time,” and “**this could lead to a false association of effects**” (Supplement 2, p.1).
- Dahl et al. (2015), although positioned somewhat outside the field of CoNS, encountered taxonomic difficulties that we feel are instructive to our concerns. In referring to their three-family classification proposal, offered a similar disclaimer regarding their inability to devise orthogonal categories: “**many practices contain elements of all three families**,” and “given the complexity of each practice listed here, we present this system as an initial step in the long process of studying the diversity of meditation practices” (p. 517).

A comprehensive review of these and several other representative taxonomy publications (nine in total) revealed several in-common themes or elements that could account for these difficulties^2^:

- Only one of the papers made any mention of taxonomic principles and theory, and all relied on the aforementioned empirical/intuitive approach.
- All used a phenetic-like method to construct overarching classification categories based on observable characteristics/elements of the various MMs.
- All but one devised their own unique first-person terminology for the construction of some or all of their classification categories.
- All but one devised their own unique first-person terminology for various other descriptive and or explanatory purposes within their classification scheme.

We concluded that the recurring nature of the problems and obstacles described above called for some kind of standardized approach that could serve as a useful tool for meditation researchers interested in avoiding these confounding taxonomic issues.

## 4. The four criteria: Guidelines for constructing and evaluating meditation classification systems for contemplative neuroscience research

Our initial review of the Philosophy of Taxonomy literature (Lubischew, 1969; Mayr, 1969; Ereshefsky, 2000) provided the theoretical and operational principles that informed our 2013 taxonomic approach. Further review for this iteration (Walsh, 2006; Ereshefsky, 2007; Austin, 2017; Kendig and Witteveen, 2020) indicated that it would be beneficial to have a criterion-based set of standards for constructing meditation classification proposals. The following Four Criteria Model was developed to facilitate a standardized and compartmentalized explanandum for our new Three Tier Classification System presented in this paper; and as a potentially useful method for evaluating the relative efficacy of various other classification proposals.

The four criteria contained within this model are: Theoretical Taxonomic Foundation, Orthogonality, Semantic Lucidity, and Utility.

### 4.1. Theoretical taxonomic foundation

The first criterion is quite simple, but is perhaps the most important—the fundamental taxonomic principle that all classification proposals require a cogent theoretical foundation (Mayr, 1969; Ereshefsky, 2000, 2007). Given this widely accepted tenet within the field of Philosophy of Taxonomy, it seems reasonable, at minimum, for all classification proposals to include an explicit statement of the authors’ philosophical and theoretical preferences, whatever those may be (e.g., functional vs. material essentialism, monism vs. pluralism, etc.). With this caveat in mind, classifications which are constructed simply because they seem to make sense on an intuitive basis can be seen as fundamentally unsound. For example, to state or imply that one’s proposal is an “empirical taxonomy” is an admission, by definition, that it lacks a theoretical basis. We caution that proposals which by-pass this essential criterion should be viewed with guarded skepticism.

### 4.2. Orthogonality

We argue that it stands to reason that all classifications of MMs intended for scientific investigation should present an effective methodology for differentiating, segregating, and grouping MMs. For the purposes of this paper, we consider orthogonality to include:

- A system of mutually exclusive overarching categories/domains whereby all MMs that are essentially and demonstrably similar are segregated together in the same domain (differentiating them from all other MMs that have not been included), and all members within any one particular domain cannot overlap and appear in any of the other domains.
- A collectively exhaustive scheme whereby the totality of the members within all of the overarching domains should encompass, at least in principle, the entire range of MMs within the sphere of interest. Creating “escape-hatch” categories such as “Other” or “Miscellaneous,” as some authors have suggested, could be considered to be a hedge and an implicit admission that there is a fundamental conceptual problem with the universality of their proposal.

### 4.3. Semantic lucidity

All classification proposals should be relatively easy to understand, and demonstrate a clarity of terms and categories that we would like to refer to here as “semantic lucidity.” For example:

- Use of clear and unambiguous terms for the labeling of categories and for the presentation of arguments; as opposed to the use of verbiage that makes it difficult to comprehend what is being proposed and how to implement it.
- Avoidance of ineffable (difficult to measure) designations and first-person neologisms/fabrications which are prone to conflation and debate; such issues arise with terms like “awareness of awareness,” “deity merging,” or “experiential fusion” which are difficult if not impossible for CoNS researchers to operationalize for scientific experimentation (see section 8.1.2 below).

### 4.4. Utility

We argue that it is reasonable to expect plausible, and readily testable categories that could be validated or refuted by CoNS researchers. In other words: can scientific investigation validate that those MMs which have been classified within a given domain demonstrate similar neurophysiological and phenomenological parameters to each other, that are significantly different than the parameters of those MMs that have been classified in other domains?

- It should be noted that by “utility,” we mean a broad conception that encompasses verifiability, testability, and plausibility for CoNS researchers, as well as the notions of usefulness, applicability, relevance, and practicality.
- The proposal should contribute toward a greater understanding of the phenomenological and/or neurophysiological elements of the meditation process in ways that are measurable and replicable.
- CoNS researchers should be able to use the proposed classification scheme to inform and test their own hypotheses.
- The proposal should not be not overly complex, convoluted, and unwieldy.

In the following two sections, we re-visit the foundational concepts presented in the NNA 2013 paper that remain central to this iteration, and we demonstrate how we applied the aforementioned Four Criteria to the construction of our new Three Tier Classification proposal.

## 5. Philosophical and conceptual bases

### 5.1. Theoretical taxonomic foundation: Applying the principles of functional essentialism

To review, the employment of an Aristotelian teleological approach requires the discernment of the functional essence of the entity/process of interest, i.e., what is its purpose?, what was it designed to do?, and We used the following set of observations, conditions, and assumptions to make this determination for MMs (NNA 2013, pp. 6–7):

- Meditation methods were derived for specific aims/purposes/goals.
- Such aims typically include various “lofty” ultimate goals and claims, such as: controlling the mind, purifying the mind, purifying the heart, gaining a greater sense of well-being, attaining spiritual insight, realizing self-actualization and enlightenment, etc. Practically speaking, these ineffable qualities (aka “traits”—a technical term used by CoNS researchers to refer to characteristics that are acquired as a result of following a meditation practice/regime over a long period of time); they are difficult to accurately describe in words or define in scientific terms, and therefore are not amenable to measurement and verification in a CoNS laboratory setting. In addition, the heterogeneity of MMs employed by long-term meditators presents a confounding factor for researchers (Matko et al., 2021). For these reasons, long-term goals were excluded from consideration in favor of explicitly stated short-term goals.
- Meditation methods were derived to facilitate a shift from the mundane waking state to a meditative state, aka an enhanced mental state (EMS) with the understanding/hope that consistent, long-term practice will lead to the attainment of a particular ultimate goal(s).
- Meditation methods exhibit a great degree of variance, e.g., how and when instructions are given, the specific techniques employed, the suggested posture, the sequence of steps, the length of time suggested, etc.
- The successful attainment of the desired EMS ultimately depends on a host of factors, both extrinsic (unpredictable outside conditions and circumstances), and intrinsic (the practitioner’s experience and expertise, motivation, current state of mind and body, etc.). These factors undoubtedly affect the quality of the experience regardless of what any given MM purports or intends to accomplish. It is reasonable to conclude therefore, that the MM is just a facilitative tool, which only offers the potential (not a guarantee) for the attainment of the immediate and ultimate goals.
- From a phenomenological perspective, the meditative state is somewhat ineffable, in that the content and qualitative nature of the experience itself often escapes description in words.

**Figure 1:**
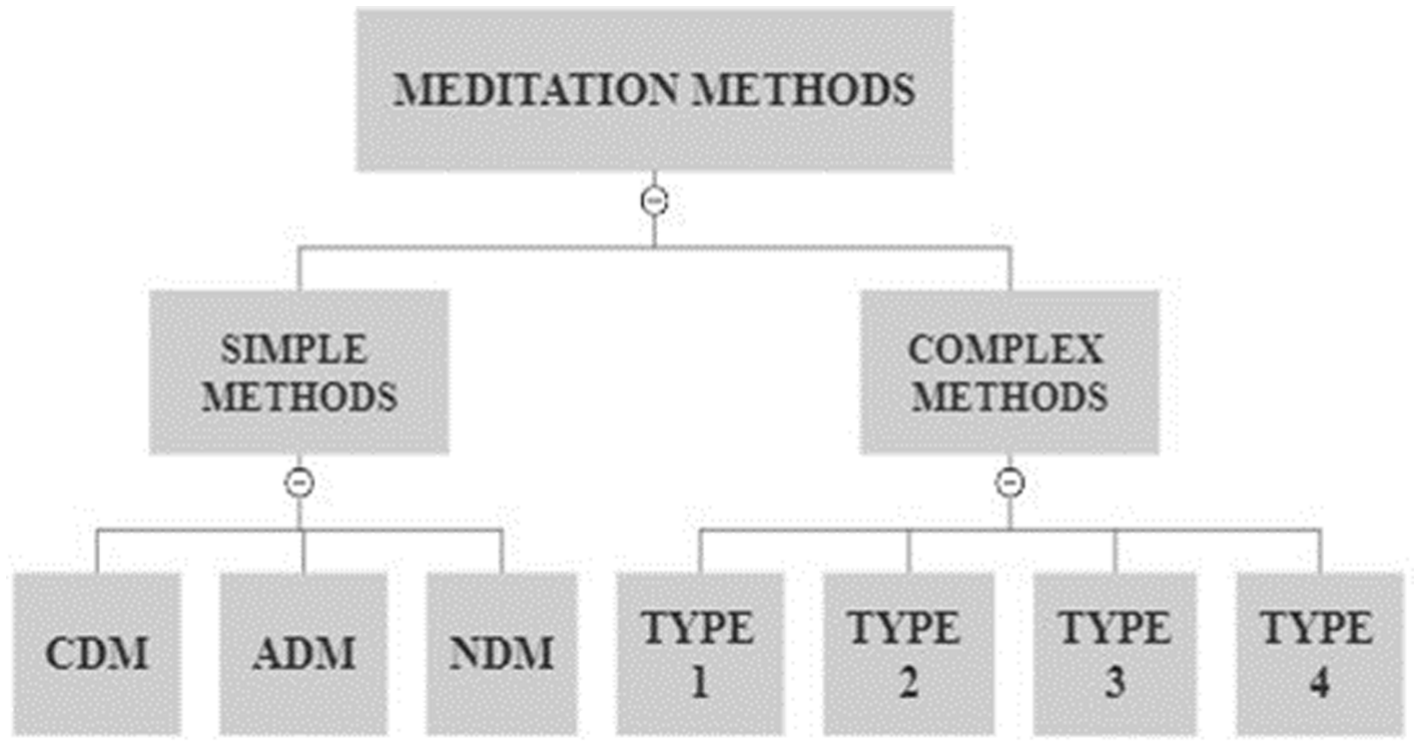
Tier 1 and Tier 2 sub-categories of the “Universe” of Meditation Methods.

For these reasons, we determined that the functional essence of a MM was its intention (purpose/goal) in an immediate sense—that is, to engender the attainment of one or more particular EMSs during a given meditation session. Long-term goals, the phenomenology of resultant states, and the peculiarities of the techniques employed were excluded from consideration for reasons outlined above. Since it may be considered rather awkward and inappropriate to think of methods as having intention, the analogous notion of directionality was used instead (Figure 1).

This assertion that MMs possess a specific intended outcome, purpose, or goal is not a new idea, and has been discussed at length in the CoNS literature since 1987 (e.g., West, 1987; Koshikawa et al., 1996; Lehmann et al., 2001; Carter et al., 2005; Hankey, 2006; Lutz et al., 2007; Ospina et al., 2007; Woods et al., 2022).

### 5.2. Key foundational concepts and semantics

In order to operationalize this notion of directionality, our 2013 paper presented a scheme and nomenclature of orthogonal domains by integrating the following foundational concepts:

#### 5.2.1. Affect and cognition

Since the rigors of scientific investigation require confidence in the validity and reliability of orthogonal distinctions, we felt that it was essential to create a nomenclature based on a stable and well-established third-person paradigm, rather than attempting to invent an entirely new and original semantic based on first person experiences or other ineffable concepts that cannot be operationalized with any clarity. Given our decision to abide by the definition of meditation as a form of mental training (see 3.2.1), it seemed most prudent to look to the Cognitive Sciences for an appropriate conceptual framework. The Cognitive Sciences tell us that all mundane waking state conscious mental activity can be reduced to affective content and processes, cognitive content and processes, and an interplay between the two (Muncy, 1986; Forgas, 2008). The notions of Affect and Cognition offered us several useful features: they have been supported by a large body of scientific research for the better part of two centuries; they have gained consensus as valid third-person designations by the philosophical, psychological, and scientific communities; and they allowed us to avoid the well-documented difficulties associated with first-person designations.^3^ For these reasons, we hypothesized that all MMs would foster meditative states that would reasonably fall into one of these two categories of mental activity—that is, mental states in which the subjective and neurophysiological correlates were either predominately affective or predominately cognitive. However, upon further research and analysis we found it necessary to modify our original supposition to include a third category—a state which was both non-affective and non-cognitive which, for the sake of brevity, we dubbed the “null state”—see section 5.2.2.

Western 18th century philosophers divided psychology’s subject matter into three distinct mental faculties: cognition, affect, and conation (Muncy, 1986; Forgas, 2008). Although the original notion of conation was given considerable attention at that time, it fell out of favor in modern cognitive science (Reitan and Wolfson, 2000), and therefore conation was not integrated into this proposal.

We relied on the standard, commonly-used definitions of affect and cognition that could be found in psychology textbooks and popular dictionaries:

- Affect: a mental state in which the phenomenological content is primarily an emotion or “feeling.”
  This thesis limits the broad spectrum of the affect paradigm to those positive emotions that are characteristic of traditional MMs, e.g., loving-kindness, compassion, and sympathetic joy (as featured in Buddhist tradition).
- Cognition: includes a host of mental processes associated with thinking; including, but not limited to: learning, reasoning, observing, perceiving, remembering, concentrating, imagining, processing information, and acquiring knowledge. With regard to traditional MMs, cognition can be exemplified by notions such as one-pointedness, mindfulness, insight, etc.

We think it is important to emphasize that when the terms “affect” and “cognition” were used in our original typology, we did not assume or imply that these mental states were “pure” and mutually exclusive (since cognitive states involve some affective components and affective states involve some cognition). Regardless, this issue was not of primary concern to us at that time because our taxonomic model was based on the intention/purpose of Simple MMs (those methods which were designed with a singularity of purpose, see section 6.1), not on the phenomenology, “purity,” or qualities of the resultant state.

#### 5.2.2. The meditative state—three kinds of enhanced mental states

In 2013, our review of the various types of MMs led us to conclude that there was a group of methods that were neither affective or cognitive in content or intention, and thus a third category was required. As such, our original proposal advanced the thesis that the meditative state consisted of three types of EMSs—an enhanced cognitive state, an enhanced affective state, and an enhanced mental state devoid of cognitive or affective content—which we dubbed “the null state.” This section focuses on the mental/phenomenological characteristics of these three states, and section 7.0 discusses their distinctive neurophysiological parameters.

- Enhanced Affective State (EAS): a meditative state in which the phenomenological content is primarily an intensified/heightened emotion or feeling. It is typically described by words, such as intense bliss, peace, love, compassion, joy, and happiness. This state is perhaps best exemplified by the Buddhist *metta* and *karuna* meditations, whereas “an unconditional feeling of loving-kindness and compassion pervades the whole mind as a way of being, with no other consideration, or discursive thoughts” (Lutz et al., 2008 p.1). Although these examples are not considered as emotions in traditional Buddhist philosophy, they can be considered as affect when interpreted into Western/English “mental typologies” (Dreyfus, 2002).
- Enhanced Cognitive State (ECS): a meditative state in which the phenomenological content is primarily an intensified/heightened cognition; exemplified by the traditional Buddhist methods of *samatha* (concentration, i.e., maintaining one’s focus of attention on a single object over a considerable period of time; Carter et al., 2005 p. 412, and *vipassana*; mindfulness and insight).
- Enhanced Null State (ENS)^4^: an enhanced mental state characterized by the absence of both affective and cognitive content, and therefore dubbed as “null.” A lengthier discussion/explanation is required here due to the relative ineffability of this denotation when compared to the distinct and well-defined notions of Affect and Cognition. ENS can be reasonably associated with various concepts that have been articulated in traditional Hindu and Buddhist canon, e.g., Sanskrit terms such as *samadhi, shunyata*, and *turiya*; and the Pali word *nirodha-samāpatti*. There have been numerous attempts to translate and explicate these arguably ineffable concepts into English, such as (in alphabetical order): “absolute unitary being” (Newberg and d’Aquili, 2001); “cessation” (Griffiths, 1986); “contentless” (Srinivasan, 2020; Winter et al., 2020); “emptiness” (Armstrong, 2017); “neutrality” (Paoletti and Ben-Soussan, 2020); “non-dual awareness/NDA” (Josipovic, 2010, 2019); “nothingness” (Powell, 1994, 1996); and “pure consciousness” (in the *Upanishads* transl. by Prabhavananda and Manchester, 1948; Maharishi Mahesh Yogi, 1986; Travis and Pearson, 2000).
  It is admittedly difficult to distill the essence connoted by all of these various esoteric terms into a singular lucid delineation. However, for the purposes of this typology, this notion of a null state is inclusive of all of the aforementioned, and is best captured by the following quotes from meditation masters, practitioners, and scholars:

1. From a highly experienced meditator (>50,000 h.) who was a subject in a recent study: “clear, aware openness without any thoughts, physical, or sensory perception, and even without any sense of self, time, and space” (Winter et al., 2020).
2. On the nature of *samadhi* and *turiya*: “no object in the mind, no content…, not meditating upon something, but dropping everything (so that) not even a ripple arises in the lake of your consciousness” (Osho, 2003); “a merging into a state of nothingness accompanied by a loss of sense of Self and duality” (by Sri Nisgaradatta in Powell, 1994); a “complete stillness, where there is no thought in the mind” and “our sense of individuality melts away” (Muktananda, 1978, 1980).
3. On the nature of *nirodha-samāpatti*: “a kind of meditation designed to exclude unwanted stimuli from awareness, to reduce the content of consciousness, and ultimately to issue in a state in which the mind has no content whatever, in which all sensory input, all perception, and all cognition and intellection have come to a complete halt” (Griffiths, 1981 p. 607); a state of “cessation” arrived through meditation “that is devoid of all mental events” (by Buddhaghosa in Griffiths, 1986 p.155).

## 6. Codifying foundational concepts into an orthogonal three tier classification system: Methodology and nomenclature

This section demonstrates how we applied the taxonomic principles of functional essentialism and orthogonal monism to classify MMs of concern to CoNS researchers. We use these principles to segregate MMs into orthogonal categories/domains within the first two Tiers of our scheme; and use a phonetic approach in the form of taxonomic keys (as the third Tier) in order to further differentiate MMs that have been grouped together in the same second Tier domain.

### 6.1. Tier 1

Given our premise that the functional intention of any given MM is to engender one or more particular EMSs within a given meditation session (see section 5.1), it seemed self-evident to divide the “universe” of MMs in Figure 1 as follows:

- Simple MMs are those methods which were designed with a singularity of purpose—to engender one of the three EMSs (described above) during a given meditation session. Simple MMs are exemplified by the traditional forms of meditation that have been the primary focus of CoNS research to date.
- Complex MMs are fundamentally different than Simple MMs in that they are intended to foster more than one type of EMS within a given meditation session, i.e., they were designed to engender a combination of separate and distinct affective, cognitive, and null states. Complex MMs present researchers with the difficult challenge of discerning which type of EMS is present at any given phase of the meditation session, and are therefore more difficult to study and explain when compared to Simple MMs. Since these methods have been mostly ignored by CoNS research (Matko et al., 2021 pp. 21–22), there is a dearth of information available about them. As such, the remainder of this paper will focus primarily on the classification of Simple MMs, although some discussion and examples are presented for Complex MMs as well.

### 6.2. Tier 2

Merges the notions of Affect and Cognition, directionality, and the three types of EMS into a scheme for further subdividing Simple MMs into orthogonal categories, and for dividing Complex MMs into four types based on a deductive exhaustion of combinations.

#### 6.2.1. Simple MMs

Simple MMs are segregated into one of three domains according to their directionality within the Affect and Cognition paradigm—Affective Directed Methods (ADM), Cognitive Directed Methods (CDM), and Null Directed Methods (NDM)

- ADMs are those MMs which are primarily purposed/intended to engender an enhanced affective state (EAS as described above) during the meditation session eg. ADM → EAS, whereas the arrow represents intention.
- CDMs are those MMs which are primarily purposed/intended to engender an enhanced cognitive state (ECS as described above) during the meditation session eg. CDM → ECS, whereas the arrow represents intention.
- NDMs are those MMs which are primarily purposed/intended to engender an enhanced null state, which is neither affective or cognitive, (ENS as described above) during the meditation session eg. NDM → ENS, whereas the arrow represents intention.

#### 6.2.2. Complex MMs

Complex MMs can be logically segregated into four categories/types based on the four possible combinations of separate EMSs intended by the method:

- Type 1 are those MMs that were purposed to engender a separate affective EMS and a separate cognitive EMS during the meditation session (not necessarily in that order).
- Type 2 are those MMs that were purposed to engender a separate affective EMS and a separate null EMS during the meditation session (not necessarily in that order).
- Type 3 are those MMs that were purposed to engender a separate cognitive EMS and a separate null EMS during the meditation session (not necessarily in that order).
- Type 4 are those MMs that were purposed to engender all three EMSs during the meditation session—separate affective, cognitive, and null EMSs (not necessarily in that order).

#### 6.2.3. Assigning MMs into appropriate Tier 2 categories/domains

An obviously crucial step in the classification process is deciding which MM goes where. Since this taxonomy is based on the intention/directionality of the method, a determination needs to be made as to the intention/goal/purpose of each MM. For the purposes of this paper, we made the following assignments based on a review of the relevant literature, public-facing claims made on official websites, personal communications with experienced practitioners, and the authors’ long-term personal practice and research experience with several of these methods.^5^

##### 6.2.3.1. Simple MMs – some examples of domain assignments

The classification of several simple meditation methods into the three main domains is displayed in Table 1.

**Table 1:** The classification of several simple meditation methods into the three main domains.

CDM	ADM	NDM
*Anapanasati* (concentration on the breath)	Theravada *karuna* and *metta* meditations	TM
Walking mindfulness meditation	Bodhisattva of Compassion man tra and chanting	*So’ham japa*
Various forms of *vipassana*	Tibetan “non-referential compassion”	*rigpa* and *mahamudra*

##### 6.2.3.2. Complex MMs

Although there is scant information about this category of MM, it is an important designation from a conceptual point of view. Here are two likely examples and a tentative classification assignment:

- The Kirtan Kriya Method is perhaps the most well-known MM which could reasonably be considered to be a Complex Type 4 Method. Kirtan Kriya (KK) was introduced to the West by Yogi Bhajan in the late 1960s—early 70s. Proponents claim it to be a traditional form of Kundalini Yoga of Northern Indian origin, taught by a lineage of Sikh Masters for over 500 years. There are many forms of KK meditation, typified by the online “12-min technique,” which has been the subject of several neuroscientific investigations (Khalsa et al., 2009; Wang et al., 2011; Moss et al., 2012; Black et al., 2013). Based on the claims made by two KK organizations on the net (www.3ho.org; www.kundaliniresearchinstitute.org)—it appears that the method is intended to engender three different EMSs during a given meditation session: “cognitive benefits” (e.g., ECS), “total stillness” (e.g., ENS), and “bliss consciousness” (e.g., EAS).
- The Light and Sound Method was purportedly derived from Sufi and Sikh traditions, and was popularized in the West by two Vietnamese meditation Masters (Master Ruma, and Master Ching Hai); however, this MM can be considered to be somewhat of an outlier since no neuroscience studies could be found. This MM aims to foster two meditative states in one session, and as such is a likely candidate for a Type 3 Complex Method. The “Light” portion of the technique involves the silent repetition of a sequence of five mantras with the intention of engendering an ENS (normal duration 1–1½ hours); it is followed by the “Sound” portion which involves concentration on one’s internal “heavenly sound” in order to facilitate an ECS of profound one-pointedness (normal duration approx.1 h).

Examples of other possible Complex MMs are: Yoga Nidra (Zaccaro et al., 2021), Sahaja Yoga, Shoonya Isha Yoga, and Raja Yoga (Panda et al., 2016). Further investigation is required.

### 6.3. Tier 3: Employs nine taxonomic keys to differentiate MMs that have been classified together within the same domain

In order to complete the classification process, a standardized method of lower-order differentiation is required to distinguish between similar MMs that have been grouped together into the same second Tier domain. To that end, our 2013 paper introduced a system of Taxonomic Keys^6^ (a methodology advanced in Mayr’s *Principles of Systematic Zoology*, 1969)—a phenetic delineation of nine salient elements of MMs, commencing with a general description of the particular MM being considered. Our keys also highlight various neurophysiological correlates associated with these elements that might otherwise be conflated with the correlates of the meditative state itself—see the notations below each key (italics are excerpted from NNA, 2013).

- General description mentions the specific name of the technique (in English, and native language if appropriate) and a general reference to the history, origin, culture, and contemplative tradition if there is one. It is also important to specify any particular style or subset, because several MMs may share the same generic name but may be significantly different, e.g., Yoga, Zen, Tai Chi, Qigong, and *Vipassana* have many forms.
- The specific keys are based on common characteristics readily observable during the meditation process and/or within the instructions associated with the MM.

#1. The specific cognitive strategies which are prescribed within the MM directions (what one has to do in order to achieve the intended result) e.g., concentration/focused attention, passive observation without attachment, visualization and imagination, memorization and repetition, selective or effortless awareness, contemplation, introspection, inquiry, sensory perception(s).

*This element is important from a research perspective since there could be distinct changes observed in the brain and body depending on the strategy used*. *For example, a visualization task is likely to activate the visual cortex, whereas reciting a prayer or phrase is likely to activate the verbal centers of the brain* (Newberg et al., 2003, 2010; Peres et al., 2012).

#2. The conceptual and/or physical object(s) that are the focus of attention, e.g., the breath, a mantra, a symbol, an image, a phrase, an idea, a narrative, a sound, etc.

*Researchers need account for the possibility that focus on certain objects might produce distinctive neurophysiological effects in the brain,* e.g.*, activation of visual centers by simple, complex, color, or black and white images, or the activation of the auditory pathways by listening to music or a voice guiding the meditation process*.

#3. Whether the MM requires certain beliefs or special knowledge, i.e., a particular religious, spiritual, metaphysical, or philosophical teaching or system.

*While this is more difficult to identify from a neurophysiological perspective, several studies have explored the various neural manifestations of different beliefs, especially between those who are believers and non-believers* (Harris et al., 2009).

#4. Whether the MM requires that the eyes remain closed or open, and if particular eye movements are prescribed.

*The visual cortex is activated when the eyes are open, especially when observing a complex scene*. *In addition, studies have shown that there are different EEG findings* (*e*.*g*.*, increased alpha power*) *during meditation practices such as Qigong between having eyes open and eyes closed* (Henz and Schöllhorn, 2017).

#5. Whether the process requires a relatively static position or certain kinetic elements. Here “static” refers to a stationary body but not necessarily an immobile body, e.g., bodily movements occur but the body still remains essentially in one place, as when the meditator changes postures from an upright sitting position to a more reclined position, or experiences involuntary jerking motions. “Kinetic” refers to prescribed movements of the body such as movements of the extremities as in walking meditation, Tai Chi, and mudras (hand movements).

*These elements are likely to effect neurophysiological changes in those parts of the brain associated with motor activity/body movement,* e.g.*, the motor cortex, basal ganglia, and cerebellum*. *In addition, movement can be associated with differences in energy utilization, adrenal function, cardiovascular function, and respiratory function*.

#6. Whether the process is non-verbal (silent/sub-vocal), verbal (vocal), or both.

*Vocalization is associated with specific cortical activity,* e.g.*, the auditory cortex and the thalamus may be differentially activated in the presence of sound*. Regarding silent mantra vs. vocal chanting, Brandmeyer et al. (2019) stated that “accumulating research suggests that silent mantra meditation may produce unique neural correlates as a result of subvocalization and/or the effect of imagining a word or phrase (see Lazar et al., 2000; Fox et al., 2014; Tomasino et al., 2014).”

#7. Whether a specific type of postural position is suggested or required, e.g., seated in a normal comfortable position, straight spine, lotus position, fully reclined, supine, or standing (this key could be considered as a sub-set of #5 above).

*The brain may respond differently to being in, and maintaining, different postures*. *Proprioceptive functions are likely to be activated as the brain works to ensure that a posture is maintained*. Research on the effects of positional changes has demonstrated decreases in EEG power in various brain centers as subjects move from upright to supine, as well as changes in the activity of the autonomic nervous system as measured by changes in skin conductance levels (Thibault et al., 2014; Travis et al., 2020).

#8. Whether the process is intrinsic (self-reliant/independent), extrinsic (dependent on the intervention or guidance of an outside person or medium), or a combination of the two.

*Performing meditation under one’s own volition* vs. *being guided can result in substantial differences in brain function*. *Evidence suggests decreased frontal activity during externally guided word generation compared to internal or volitional word generation* (Crosson et al., 2001).

#9. Whether there are any specific recommendations for type or control of breathing, or whether a normal breathing pattern is to be maintained.

*Breathing, especially when controlled, can result in specific changes in brain and body physiology*. *Controlled breathing may alter heart rate, blood pressure, and metabolism while also changing the function of the brain* (Floyd et al., 2003; Barnes et al., 2011).

#### 6.3.1. How taxonomic keys are used to differentiate simple MMs that have been classified within the same domain: Two examples

The following two MMs are both classified as CDMs,^7^ however when the taxonomic keys are applied it can be seen that only four of the keys are similar (#3,6,8,9 in *italics*); and five are different (in bold standard print).

- *Anapanasati* (concentration on the breath) is one of the most popular and most researched of the traditional Buddhist techniques; in its most basic form is considered to be a *samatha* MM, intended to calm and focus the mind.
  1. **utilizes concentration as the main cognitive strategy**;
  2. **the focus is on the breath and the parts of the body associated with breathing**;
  3. *knowledge and belief in the teachings of Buddhism is recommended but not essential;*
  4. **the eyes are closed**;
  5. **the body is static**;
  6. *the practice is mostly non-verbal, although out-loud/verbal versions are also employed*;
  7. **the recommended posture is seated with straight spine; a cross-legged/lotus position is optional**;
  8. *the solo practice is intrinsic, but an extrinsic guided form is common in group practice*; and
  9. *normal breathing pattern with no intention to control the breath*.
- Walking meditation is a simple, well-known technique, commonly taught and practiced at Buddhist monasteries and at meditation retreats all over the world; exemplified here by the technique taught by the famous Vietnamese meditation Master Thich Nhat Hanh, as a means to cultivate mindfulness (e.g., “Peace is Every Step,” 1991).

1. **utilizes awareness as the main cognitive strategy**;
2. **the focus of attention is on the feet and the sensations of walking**;
3. *knowledge and belief in the teachings of Buddhism is recommended but not essential*;
4. **the eyes are open**;
5. **the body is in motion (kinetic), walking in a slow but normal gait, preferably bare-footed**;
6. *non-verbal*;
7. **standing position with a normal, comfortable posture**;
8. *intrinsic*; and
9. *normal breathing pattern with no intention to control the breath*.

## 7. Supportive neuroscientific findings

As previously discussed, our 2013 publication advanced the thesis that all MMs are causally related to one of three resultant meditative states, which we described as enhanced mental states (EMS). That paper cited research from the broader fields of cognitive and affective neuroscience, as well as from the field of CoNS, which supported the notion that these enhanced cognitive, affective, and null states demonstrated distinctly different and measurable neurophysiological correlates (e.g., Lehmann et al., 2001; Dalgleish, 2004; Carter et al., 2005; Cahn and Polich, 2006; Hankey, 2006; Holzel et al., 2007, 2008; Lutz et al., 2007, 2008; Davidson, 2010; Travis and Shear, 2010; Josipovic et al., 2011; Leung et al., 2013).^8^

Since then, our original thesis has been supported by more recent research as well (e.g., Dahl et al., 2015; Brandmeyer et al., 2019; Josipovic, 2019; Raffone et al., 2019; Afonso et al., 2020; Yordanova et al., 2020, 2021). Lee et al. (2012, p.7) concluded that “different forms of meditation have meditation-specific effects on neural activity, rather than a common neural mechanism”; “different forms of meditation practice create domain-specific plastic changes in neural activity”; and “each form of meditation is associated with a dissociable pattern of neural activity.” In a review of meditation research findings to date, Travis concurs: “the assumption that a common brain marker would emerge by combining different meditation practices together in one analysis is flawed. Meditation procedures differ. Some meditations involve deep concentration, others prescribe attention to external and internal stimuli and others are inwardly directed towards nondual states. Thus, brain patterns from different practices would not be expected to converge to a common pattern.” (Travis, 2020 pp. 3–4). It seems fair to say, that it has become a widely-accepted premise within the field of CoNS that different types of MMs demonstrate different neurophysiological correlates.

On the basis of these and other similar findings, we can confidently state that during meditation, neural activity in the brain (i.e., connectivity between different nodes of functional networks, and/or higher activation in certain brain regions) varies dependent upon which type of MM is employed. For example, CoNS research has demonstrated that the EAS engendered by various ADMs (e.g., loving kindness and compassion MMs) demonstrates distinctly different neural activity than the ECS engendered by various CDMs (e.g., mindfulness and concentration MMs). In addition, cognitive and affective neuroscience research has demonstrated specific hormones and neural transmitters that are distinctive for affective vs. cognitive states (Dalgleish, 2004; Ali et al., 2018). Regarding NDMs, they have been shown to engender not only distinctive neural activity in particular brain regions and circuitry, but also deactivations in certain regions which are unique to this category (Lehmann et al., 2001; Newberg and Iversen, 2003; Travis, 2020; Winter et al., 2020). Researchers have also noted distinctive EEG patterns associated with different meditation methods. For example, participants in a Buddhist meditation retreat were observed to have decreased alpha frequency across meditation retreats and in direct relation to the amount of meditative practice (Saggar et al., 2012). Transcendental meditation has been associated with increases in the frequency of peak EEG power (Travis and Pearson, 2000). Importantly, Yordanova and colleagues showed that there were similarities and differences between three different practices—focused attention, open monitoring, and loving kindness meditation (Yordanova et al., 2020, 2021). All meditation practices were associated with a common connectivity pattern characterized by increased connectivity of broadly distributed delta networks, left-hemispheric theta networks, and right-hemispheric alpha networks. However, the meditation practices were differentiated on the basis of left or right lateralized beta networks.

It is not the intention of this paper to undertake and present an exhaustive review of CoNS research or the disciplines of cognitive/affective neuroscience. Rather, the following section presents a sampling of research findings, in greater detail, which support the conclusions cited above.

### 7.1. Cognition: The ECS

It is reasonable to expect that an ECS engendered by a CDM would demonstrate heightened neural activity in one or more of the cortical areas of the brain (and/or changes in connectivity between different nodes of functional networks) that subserve higher cognitive processing, e.g., attention, concentration, observation, verbal reasoning, and abstract thought. Since the early 1990s, a number of neuroimaging studies have corroborated this hypothesis by showing activation of the PFC during CDMs (Herzog et al., 1990-1991; Lou et al., 1999; Lazar et al., 2000; Brefczynski-Lewis et al., 2007; Lutz et al., 2008); and activation of the broader neural circuitry of the “executive attention network” (Holzel et al., 2007). More recently, meditation researchers using fMRI have identified specific neural networks associated with CDMs, e.g., the central-executive network (Lutz et al., 2015); and the default mode network (Brandmeyer et al., 2019). In addition, EEG research of CDMs has demonstrated distinctive patterns of neural activity, e.g., higher occipital gamma, frontal midline theta, and somatosensory alpha (Travis, 2020).

### 7.2. Affect: The EAS

Affective neuroscience research has long established a connection between affect and specific areas of brain function, e.g., the thalamus, hypothalamus, cingulate gyrus, hippocampi, and amygdala (Dalgleish, 2004; Armony, 2013; Celeghin et al., 2017). CoNS researchers have been able to distinguish affect from other mental states by its distinct and measurable subjective and neurobiological correlates (e.g., Davidson and Irwin, 1999; Lutz et al., 2004, 2008; Hanson and Mendius, 2009; Davidson, 2010; Travis and Shear, 2010; Lee et al., 2012; Leung et al., 2013; Mascaro et al., 2013; Dahl et al., 2015; Engen and Singer, 2016).

### 7.3. The non-cognitive/non-affective null state: The ENS

Since the ENS associated with NDMs presents a greater challenge to describe and measure (see section 5.2.2), this null state warrants a bit more discussion here than the more straightforward categories of Affect and Cognition. CoNS research of MMs which seem to fall into this non-affect/non-cognitive category include: TM (e.g., as described by Travis and Pearson, 2000): non-dual awareness (NDA) methods (e.g., as described by Josipovic, 2010); and *So’ham japa* as described by Kumar et al. (2014).

- Since TM purports to engender a state of “pure consciousness” (PC), we have classified it as a NDM. TM research has reported, among other things, that frontal alpha1 EEG coherence is distinctive of TM practice vs. other MMs (Travis and Shear, 2010; Travis et al., 2020). Hankey (2006) reported psychophysiological correlates for PC during TM that were distinctly different than one-pointed and compassion techniques; and the TM research conducted by Travis and Pearson (2000) reported distinct changes in sympathetic and parasympathetic measures during subject reports of PC.
- NDA is included here because it is described as an enhanced state which is profoundly different than the mundane waking state—characterized by a dissolution of the subject-object sense of duality, with an absence of both affective and cognitive mental content (Josipovic, 2019; Josipovic and Miskovic, 2020). Examples of NDA meditations include: *rigpa* and *mahamudra* meditations from the Tibetan Buddhist tradition, various Kashimiri Shaivist meditations, Zen *shikan-taza,* and various other meditations in the Vedanta tradition (Josipovic, 2022 personal communication.).
  Josipovic has proposed that a dynamic functional network with its main node in the central area of precuneus, and its main axis node in the dorso-lateral prefrontal cortex, is the likely neural correlate of NDA (Josipovic, 2013); and he hypothesized that the absence of significant changes in connectivity of the dorsal anterior cingulate cortex (ACC) may be indicative of the less cognitively controlled nature of this style of meditation (Josipovic et al., 2011).
- The *So’ham* MM (of ancient Hindu Vedanta origin) is included here because proponents claim that the method can engender a null state of *samadhi* (Muktananda, 1978, 1980; Shankarananda, 2003). Kumar et al. (2014) conducted an analysis of long-term *So’ham* meditators and found significantly higher gray matter density in the dorsal brain stem, left ventral pallidum, and left supplementary motor area compared with age-matched non-meditators. They also detected different changes in brain structure when compared with other forms of meditation.

In addition to these studies, various other CoNS research projects have posited the possibility of deafferentation/deactivation/dampening of cognitive and affective areas of the brain during the ENS (Herzog et al., 1990-1991; Newberg et al., 2010; Travis, 2020; Winter et al., 2020).

## 8. A critical review of nine other classification proposals (2007–2022)

One of the stated objectives of this paper was to compare and contrast this proposal with other taxonomies that have appeared in the literature. Nine other “competing” taxonomy of meditation publications, by seven different lead authors, were reviewed and evaluated by applying the Four Criteria Model (see section 4.0)—Taxonomic Foundation, Orthogonality, Semantic Lucidity, and Utility.

Here is the list of the nine publications, in chronological order. Three of these did not emanate from, nor were specifically directed to, the CoNS community—Dahl et al., Ospina et al., and Pilla et al., but were included here because they were notable contributions to the broader field of meditation research and provided instructive examples of the taxonomic issues that are of concern in this paper.

1. Ospina et al. (2007). “Meditation Practices for Health: State of the Research”—see section 3.3 for additional comments about this paper.
2. Fox et al. (2014). ^9^ “Is meditation associated with altered brain structure? A systematic review and meta-analysis of morphometric neuroimaging in meditation practitioners.”
3. Dahl et al. (2015). “Reconstructing and deconstructing the self: cognitive mechanisms in meditation practice.”
4. Matko et al. (2018, 2021), Matko and Sedlmeier (2019):

- “The Top 10: Prevalence and Popularity of Basic Meditation Practices in Different Spiritual Traditions” (2018).
- “What Is Meditation? Proposing an Empirically Derived Classification System” (2019).
- “What Do Meditators Do When They Meditate? Proposing a Novel Basis for Future Meditation Research” (2021).

5. Pilla et al. (2020). “Toward a Framework for Reporting and Differentiating Key Features of Meditation and Mindfulness Based Interventions.”
6. Engström et al. (2022): “A Review of the Methodology, Taxonomy, and Definitions in Recent fMRI Research on Meditation.”
7. Sparby and Sacchet (2022). “Defining Meditation: Foundations for an Activity-Based Phenomenological Classification System.”

### 8.1. Summary of our critique using the four criteria model

Several theoretical and methodological issues plagued these proposals.

#### 8.1.1. Theoretical taxonomic foundation

Eight of the papers made no mention whatsoever of taxonomic theory, principles, or methods. We have previously discussed and emphasized why this is a critical oversight (sections 3.3 and 4.1). Only Sparby and Sacchet cited both classical and contemporary theories of taxonomy. Unfortunately, they chose to base their thesis in the spirit of material essentialism and constructed their classification categories using the phenetic notion of observable characteristics. This led to an intuitive/empirical approach and the untoward outcomes associated with this strategy (see section 3.3).

To further illustrate why a phenetic taxonomic strategy is problematic with regard to MMs, we examine two characteristics that were often employed as overarching domains in the classification proposals that we reviewed:

- Mantra: Although MMs grouped under this category undoubtedly share a common feature, it is important to ask—are all mantra methods “essentially” the same? (e.g., Ospina et al., 2007; Matko et al., 2018, 2021; Matko and Sedlmeier, 2019; Pilla et al., 2020). Although there are many MMs which proffer a mantra technique (especially those associated with Buddhist and Hindu traditional practices), one can observe marked differences between MMs within this proposed category. For example, compare the Buddhist MM which uses the mantra “*Om mani padme hum*” to facilitate an EAS of compassion vs. the TM mantra which aims to engender an ENS of “pure consciousness.” Even though both methods utilize a mantra technique, the goal is not the same, and the resultant EMS would demonstrate markedly different neurophysiological and phenomenological correlates—the former primarily affective and the later primarily null. In this case, we can see that the characteristic of mantra simply does not provide the level of distinction/differentiation required for an orthogonal overarching domain.
- Somatic elements: This term refers to various bodily/physical characteristics, such as the prescribed postures and movements in Hatha Yoga, Tai Chi, Qigong, walking meditation, and mudras (hand movements/gestures; e.g., Matko et al., 2018, 2021; Matko and Sedlmeier, 2019; Engström et al., 2022). Once again, we pose the fundamental question—is it efficacious for all so-called “somatic” methods to be grouped together in the same category, even though many of these MMs would demonstrate varying goals/intentions, and markedly different neurophysiological and phenomenological correlates? Since somatic elements are present in so many diverse MMs, they offer no possibility for orthogonal discrimination.
  In addition, it is important to recognize that somatic elements (when present) are often used to facilitate the attainment of the desired meditative state, but they are not an end-point or a primary goal. As an example, we examine the walking meditation method discussed in 6.3.1, which some authors classify as a somatic technique. Walking meditation was derived in the Buddhist tradition as an effective way to cultivate mindfulness. It is not practiced in order to improve one’s walking skills. The act of walking is simply one of many modalities/techniques that are chosen in mindfulness training to develop the faculties of awareness. Thich Nhat Hahn (1991), the famous meditation Master, not only promoted walking meditation, but he was fond of saying that washing the dishes was also one of his favorite mindfulness exercises!

Although the aforementioned characteristics of “mantra” and “somatic elements” are problematic when employed as orthogonal categories, we have found them to be quite useful within our taxonomic keys scheme—see section 6.3.

#### 8.1.2. Semantic lucidity

The Ospina team used standard, well-known terms for the construction of all of their overarching classification domains, and they were the only ones to do so. All the remaining papers devised first-person terms and neologisms for all or some of their domain designations, and for other descriptive purposes as well.

Here are some specific examples to illustrate why such choices are problematic:

- formulating classification categories based on first-person semantics and/or newly coined terminology
  When formulating terminology for their classification categories, many authors “borrow” previously published first-person semantics and/or devise their own designations, such as: “Constructive, and Deconstructive Families” (Dahl et al., 2015); “Amount of Body Orientation” and “Activation” (Matko et al., 2021); “Wholesome and Unwholesome Qualities” and “The Present Moment” (Engström et al., 2022); “Awareness of Awareness” and “Awareness of Objects” (Sparby and Sacchet, 2022). This paper has previously outlined why using these kinds of terms for this purpose is problematic—they are difficult for CoNS researchers to measure, and are wide-open to misinterpretation, conflation, and debate; especially when compared to stable and universally accepted third-person constructs such as Affect and Cognition.^10^
- using unique first-person terminology for various purposes within one’s classification scheme.
  Various authors attempted to explicate and differentiate MMs by devising their own notions such as: “let go” vs. “stay open,” “release” vs. “focus,” and “justifiable belief of primary and secondary intentions” (Sparby and Sacchet, 2022); “visualizing/expanding” and “deity merging” (Matko et al., 2018, 2021; Matko and Sedlmeier, 2019); “wishing/reflection” (Pilla et al., 2020); and “experiential fusion” and “self-schema” (Dahl et al., 2015). When authors use nebulous terms such as these within their classification schema; terms that may be considered ambiguous and difficult to measure and quantify, it becomes extremely challenging for CoNS researchers to quantify, validate, and replicate their theses. For these reasons, the scientific efficacy of their proposals can be called to question.

#### 8.1.3. Orthogonality

Of the nine publications reviewed, many used classification categories/domains that were too broad to successfully segregate and differentiate MMs, and none offered a classification system capable of orthogonal distinctions:

- Seven papers made explicit statements conceding that their attempts to construct orthogonal categories had failed or were not without problem, e.g., Dahl et al., Fox et al., three papers by Matko et al., Ospina et al., and Sparby and Sacchet—see section 3.3 for more detail.
- Pilla et al. used domain categories that were too broad or vague, e.g., “Mantra,” “Yogic,” and “Others.”
- Engström et al. also used domain categories that were too broad or vague, e.g., “Somatic,” and “Self”; and assigned many MMs to two or more of their domain categories.

#### 8.1.4. Utility

Unfortunately, none of the papers satisfied the general or specific guidelines suggested under this criterion; especially the critical importance of providing CoNS researchers with plausible, unambiguous, and readily testable classification categories.

## 9. Summary

The primary goal of this paper was to advance a cogent theoretical argument and an applicable structural model for CoNS; one which could successfully classify all applicable meditation methods into orthogonal domains. To those ends, a new Three Tier Classification System was presented based on established taxonomic principles and methods. This proposal also intended to influence due consideration of the merits of a third-person codification system based on the paradigm of Affect and Cognition vs. first-person nomenclatures and categories. We attempted to present a relatively straightforward classification scheme that could provide taxonomic order for the vast diversity of MMs that have been, and will be, the subject of study within this field. As such, the efficacy of our Three Tier Classification proposal must ultimately be determined by the CoNS community.

We attempted to implement these goals by:

- expanding our original 2013 taxonomy proposal to encompass the full range of MMs within the purview of CoNS by accounting for both Simple and Complex MMs; this distinction became the first Tier of our new classification scheme;
- reviewing the salient definitional issues regarding meditation methods and states;
- providing discussion and a glossary of relevant terms/concepts from the fields of Taxonomic Philosophy and the Science of Systematics that could be applied toward the classification of MMs;
- demonstrating why the Aristotelian teleological theory of functional essentialism was the most efficacious taxonomic approach with regard to immaterial processes such as meditation;
- presenting the rationale for choosing “intention/directionality” as the functional essence of MMs;
- explaining the rationale behind merging this notion of “intention/directionality” with the paradigm of Affect and Cognition to form the conceptual basis of our second Tier—the three distinct meditative states of EAS, ECS, and ENS, and their corresponding classification categories of ADM, CDM, and NDM. The assignment of MMs within the second Tier was exemplified for both Simple and Complex methods;
- introducing a Four Criterion set of guidelines for constructing and evaluating classification systems for MMs: Theoretical Taxonomic Foundation, Orthogonality, Semantic Lucidity, and Utility; and explaining how these criteria informed the construction of our Three Tier Classification System;
- promoting the concept of Taxonomic Keys to serve several purposes: as the basis of the third Tier of the classification scheme so as to differentiate similar MMs within the same domain using nine salient characteristics/elements; as a guide for researchers regarding the neurophysiological implications of these elements, and as a standardized protocol for describing MMs;
- citing and reviewing more than two decades of neuroscience research to support and validate the taxonomic thesis and classification scheme of this paper;
- reviewing the theoretical and methodological issues that have plagued various other taxonomy of meditation proposals; and showing by specific examples why it is ineffective to bypass established taxonomic theories and principles in favor of an intuitive/empirical approach, observable characteristics, and first-person designations.

## 10. Suggestions for future study

- Develop a much-needed standardized descriptive methodology for the full range of existing MMs of interest to CoNS researchers by expanding and refining the taxonomic keys suggested in this paper in a way that could gain consensus within the CoNS community.
- Initiate a comprehensive effort to determine, by consensus, the most appropriate classification assignments within the Affective/Cognitive/Null typology for the full range of Simple MMs; and identify, explicate, and classify a more exhaustive list of Complex MMs. This could be accomplished by soliciting the input of expert practitioners and proponents of each method; and by employing effective search, content analysis, and meta-analysis methods, e.g., the survey methodology advanced by Koshikawa et al. (1996); a standardized interview and analysis similar to the modified Delphi techniques used by Ospina et al. (2007) and Bond et al. (2009); and the sophisticated analyses employed by Matko et al., Pilla et al., and Engström et al. (see section 8.0).
- Develop a laboratory environment for CoNS research, which is more “user-friendly” and less distracting for meditation subjects *via* a broader utilization of recent technological advances in less obtrusive neuroelectric instrumentation and methods, e.g., shielded equipment that allows for simultaneous EEG and ANS recordings inside an active MRI tunnel (in order to better differentiate targeted brain activity from the confounding influences of the fMRI/MRI process itself); new MRI machines that allow the subject to sit during the recording (thereby eliminating the confound of requiring subjects to lie down during meditation); new MRI technology designed to reduce the acoustic noise output, e.g., GE Healthcare’s Silent Scan; invention of mobile, and light weight equipment to enable effective recording of those MMs which feature kinetic characteristics.
- Undertake a multi-disciplinary investigation of the phenomenology of the Null State in an effort to determine if there are fundamental orthogonal distinctions between various MMs (sub-sets) that we have classified in this domain, i.e., pure consciousness compared to *nirodha-samapatti*.

## 11. Conclusion

This project was motivated by the conviction that, in order for our field of CoNS to progress, it is essential for meditation researchers to have a valid and reliable taxonomic system with which to classify MMs, test their hypotheses, and compare their findings. As such, this proposal was intended to offer a cogent theoretical and methodological approach which highlighted the critical importance of utilizing established taxonomic principles and methods—in this case, a teleological, essentialist, third-person approach; and to shed light on some of the confounding issues associated with various other taxonomy proposals that have been published in the last 15 years.

Another emphasis of this paper, worth repeating here, is a plea to meditation researchers and authors to consider reporting more than just the name and a cursory general description of the various MMs that are being studied. The adoption of a standardized list of specific and agreed-upon taxonomic keys could provide a reasonable way to more effectively describe and compare various MMs, and to explicitly account for the neurophysiological correlates associated with these keys.

Hopefully the CoNS community will be interested in the taxonomic model and ideas that we have presented in this paper, will test the efficacy of the designations that have been proposed, and offer constructive suggestions and refinements that will ultimately lead to a classification system that is worthy of consensus.

## Data availability statement

The original contributions presented in the study are included in the article/supplementary material, further inquiries can be directed to the corresponding author.

## Author contributions

All authors listed have made a substantial, direct, and intellectual contribution to the work and approved it for publication.

## Conflict of interest

The authors declare that the research was conducted in the absence of any commercial or financial relationships that could be construed as a potential conflict of interest.

## Publisher’s note

All claims expressed in this article are solely those of the authors and do not necessarily represent those of their affiliated organizations, or those of the publisher, the editors and the reviewers. Any product that may be evaluated in this article, or claim that may be made by its manufacturer, is not guaranteed or endorsed by the publisher.

## Footnotes

^1^For the purposes of this paper, we use the term "contemplative neuroscience" as originally conceived at the Mind and Life Institute and popularized by Dr. Richard Davidson in 2012: https://www.mindful.org/the-emerging-field-of-contemplative-neuroscience i.e., *the field in which neuroscience tools* (e.g.*, fMRI, EEG, PET, and SPECT*) *are used to study the effects of meditation*. As such our proposal is addressed to the neuroscientific research of contemplative meditation methods.

^2^For a more thorough analysis see section 8.0.

^3^**F**or a more thorough discussion of the philosophical issues surrounding first-person vs. third-person perspectives and designations, and the issues related to "introspectionism" (see: Costall, 2006; Schwitzgebel, 2010; Choifer, 2018; NNA, 2013 p.3).

^4^The term "null" was chosen as a synonym for such notions as contentless, devoid, empty, nothingness, etc. To be clear, whenever the term "null" is used in the context of this paper, it refers to a state which is absent of both affective and cognitive content. Rather than use the term "non-affective/non-cognitive" to designate this state, we use "null" for the sake of brevity and convenience. Although it may be deemed awkward or paradoxical to consider a null state as being enhanced, we used that designation to connote a profound, heightened state of consciousness as compared with the mundane waking state, and for the sake of terminological consistency with the two other members of this tripartite typology.

^5^These examples and assignments should not be construed as a claim of final delineation, but rather as an illustration of how this classification scheme can be used to place MMs into suitable orthogonal categories—see Suggestions for Future Study. For more examples see NNA 2013.

^6^The use of taxonomic keys to aid in the classification procedure is not a new idea. Much of Aristotle’s scheme for the differentiation of animals was presented in the form of "simple dichotomous descriptions, i.e., bloodless animals vs. animals with blood" (Mayr, 1969 p.276). Note that the nine taxonomic keys presented here are not presumed to be all-inclusive—see Suggestions for Future Study.

^7^For eight more examples of the assignment of Taxonomic Keys to various MMs—see NNA (2013, p.10–12).

^8^For a more detailed discussion (see NNA, 2013 pp. 7–9).

^9^These authors attempted, but declined to present a classification proposal for MMs—their paper is included here because their rationale for not doing so is instructive and germane to this discussion (see section 3.3).

^10^For a more thorough discussion of the philosophical issues surrounding first-person vs. third-person perspectives and designations, and the issues related to introspectionism (see: Costall, 2006; Schwitzgebel, 2010; Choifer, 2018; NNA, 2013 p.3).

## References

[ref1] Afonso R. F. Kraft I. Aratanha M. A. Kozasa E. H. (2020). Neural correlates of meditation: a review of structural and functional MRI studies. Front. Biosci. 12, 92–115. doi: 10.2741/S542, PMID: 32114450

[ref2] Ali S. A. Begum T. Reza F. (2018). Hormonal influences on cognitive function. Malays. J. Med. Sci. 25, 31–41. doi: 10.21315/mjms2018.25.4.3, PMID: 30914845 PMC6422548

[ref3] Armony J. (ed.). (2013). The Cambridge Handbook of Human Affective Neuroscience. Cambridge: Cambridge University Press

[ref4] Armstrong G. (2017). Emptiness. Somerville, MA: Wisdom Publishers

[ref5] Austin C. (2017). Aristotelian essentialism: essence in the age of evolution. Synthese 194, 2539–2556. doi: 10.1007/s11229-016-1066-4

[ref6] Awasthi B. (2012). Issues and perspectives in meditation research: in search for a definition. Front. Psychol. 3:613. doi: 10.3389/fpsyg.2012.00613, PMID: 23335908 PMC3541715

[ref7] Bærentsen K. B. Stødkilde-Jørgensen H. Sommerlund B. Hartmann T. Damsgaard-Madsen J. Fosnæs M.. (2009). An investigation of brain processes supporting meditation. Cogn. Process. 11, 57–84. doi: 10.1007/s10339009-0342-3 19876663

[ref8] Barinaga M. (2003). Studying the well-trained mind. Science 302, 44–46. doi: 10.1126/science.302.5642.44, PMID: 14526055

[ref9] Barnes V. A. Pendergrast R. A. Harshfield G. A. Treiber F. A. (2011). Impact of breathing awareness meditation on ambulatory blood pressure and sodium handling in prehypertensive African American adolescents. Ethn. Dis. 48, 59–64. doi: 10.1016/j.jadohealth.2010.05.019, PMID: 18447091 PMC3216041

[ref10] Black D. Cole S. W. Irwin M. R. Breen E. (2013). Yogic meditation reverses NF-kB and IRF-related transcriptome dynamics in leukocytes of family dementia caregivers in a randomized controlled trial. Psychoneuroendocrinology 38, 348–355. doi: 10.1016/j.psyneuen.2012.06.011, PMID: 22795617 PMC3494746

[ref11] Bond K. Ospina M. B. Hooton N. Bialy L. Dryden D. M. Buscemi N.. (2009). Defining a complex intervention: the development of demarcation criteria for “meditation.”. Psychol. Relig. Spiritual. 1, 129–137. doi: 10.1037/a0015736

[ref12] Brandmeyer T. Delorme A. Wahbeh H. (2019). The neuroscience of meditation: classification, phenomenology, correlates, and mechanisms. Prog. Brain Res. 244, 1–29. doi: 10.1016/bs.pbr.2018.10.020, PMID: 30732832

[ref13] Brefczynski-Lewis J. A. Lutz A. Schaefer H. S. Levinson D. B. Davidson R. J. (2007). Neural correlates of attentional expertise in long-term meditation practitioners. Proc. Natl. Acad. Sci. U. S. A. 104, 11483–11488. doi: 10.1073/pnas.0606552104, PMID: 17596341 PMC1903340

[ref14] Cahn B. R. Polich J. (2006). Meditation states and traits: EEG, ERP, and neuroimaging studies. Psychol. Bull. 132, 180–211. doi: 10.1037/0033-2909.132.2.180, PMID: 16536641

[ref15] Carter O. Presti D. E. Callistemon C. Ungerer Y. Liu G. B. Pettigrew J. D. (2005). Meditation alters perceptual rivalry in Tibetan Buddhist monks. Curr. Biol. 15, R412–R413. doi: 10.1016/j.cub.2005.05.043, PMID: 15936259

[ref16] Celeghin A. Diano M. Bagnis A. Viola M. Tamietto M. (2017). Basic emotions in human neuroscience: neuroimaging and beyond. Front. Psychol. 8:1432. doi: 10.3389/fpsyg.2017.01432, PMID: 28883803 PMC5573709

[ref17] Choifer A. (2018). A new understanding of the first-person and third-person perspectives. Anom. razvitiia zarodysheĭ cheloveka 47, 333–371. doi: 10.1080/05568641.2018.1450160

[ref18] Costall A. (2006). ‘Introspectionism’ and the mythical origins of scientific psychology. Conscious. Cogn. 15, 634–654. doi: 10.1016/j.concog.2006.09.008, PMID: 17174788

[ref19] Crosson B. Sadek J. R. Maron L. (2001). Relative shift in activity from medial to lateral frontal cortex during internally versus externally guided word generation. J. Cogn. Neurosci. 13, 272–283. doi: 10.1162/089892901564225, PMID: 11244551

[ref20] Dahl C. Lutz A. Davidson R. (2015). Reconstructing and deconstructing the self: cognitive mechanisms in meditation practice. Trends Cogn. Sci. 19, 515–523. doi: 10.1016/j.tics.2015.07.001, PMID: 26231761 PMC4595910

[ref21] Dalgleish T. (2004). The emotional brain. Nat. Rev. Neurosci. 5, 583–589. doi: 10.1038/nrn1432 15208700

[ref22] Das N. N. Gastaut H. C. (1955). Variations de l'activite electrique du cerveau, du coeur et des muscles squelletiques au cours de la meditation et de l'extase yogique. Electroencephalogr. Clin. Neurophysiol. 6, 211–219.

[ref23] Davidson R. (2010). Empirical explorations of mindfulness: conceptual and methodological conundrums. Emotion 10, 8–11. doi: 10.1037/a0018480, PMID: 20141297 PMC4485421

[ref24] Davidson R. J. Irwin W. (1999). The functional neuroanatomy of emotion and affective style. Trends Cogn. Sci. 3, 11–21. doi: 10.1016/s1364-6613(98)01265-0, PMID: 10234222

[ref25] Dreyfus G. (2002). “Is compassion an emotion? A cross cultural exploration of mental typologies” in Visions of Compassion. eds. Davidson R. Harrington A. (Oxford: Oxford University Press), 31–45.

[ref26] Engen H. Singer T. (2016). Affect and motivation are critical in constructive meditation. Trends Cogn. Sci. 20, 159–160. doi: 10.1016/j.tics.2015.11.004, PMID: 26686556

[ref27] Engström M. Willander J. Simon R. (2022). A review of the methodology, taxonomy, and definitions in recent fMRI research on meditation. Mindfulness 13, 541–555. doi: 10.1007/s12671-021-01782-7

[ref28] Ereshefsky M. (2000). The Poverty of the Linnaean Hierarchy: A Philosophical Study of Biological Taxonomy. Cambridge: Cambridge University Press

[ref29] Ereshefsky M. (2007). “Species, taxonomy, and systematics” in Philosophy of Biology. eds. Matthen M. Stephens C. (Oxford, UK: Elsevier), 403–427.

[ref30] Floyd T. F. Clark J. M. Gelfand R. Detre J. A. Ratcliffe S. Guvakov D.. (2003). Independent cerebral vasoconstrictive effects of hyperoxia and accompanying arterial hypocapnia at 1 ATA. J. Appl. Physiol. 95, 2453–2461. doi: 10.1152/japplphysiol.00303.2003, PMID: 12937024

[ref31] Forgas J. (2008). Affect and cognition. Perspect. Psychol. Sci. 3, 94–101. doi: 10.1111/j.1745-6916.2008.00067.x 26158876

[ref32] Fox K. Nijeboera S. Dixona M. L. Flomanb J. L. Ellamila M. Rumaka S. P.. (2014). Is meditation associated with altered brain structure? A systematic review and meta-analysis of morphometric neuroimaging in meditation practitioners. Neurosci. Biobehav. Rev. 43, 48–73. doi: 10.1016/j.neubiorev.2014.03.016, PMID: 24705269

[ref33] Griffiths P. (1981). Concentration or insight: the problematic of Theravāda Buddhist meditation theory. J. Bible Relig. XLIX, 605–624. doi: 10.1093/jaarel/XLIX.4.605

[ref34] Griffiths P. (1986). On Being Mindless: Buddhist Meditation and the Mind-Body Problem. Delhi, India: Sri Satguru Publ

[ref36] Hankey A. (2006). Studies of advanced stages of meditation in the Tibetan Buddhist and Vedic traditions. Evid. Based Complement. Alternat. Med. 3, 513–521. doi: 10.1093/ecam/nel040, PMID: 17173116 PMC1697747

[ref37] Hanson R. Mendius R. (2009). Buddha’s Brain. Oakland, CA: New Harbinger Publishers

[ref38] Harris S. Kaplan J. T. Curiel A. Bookheimer S. Y. Iacoboni M. Cohen M. S. (2009). The neural correlates of religious and nonreligious belief. PLoS One 4:e0007272. doi: 10.1371/journal.pone.0007272, PMID: 19794914 PMC2748718

[ref39] Henz D. Schöllhorn W. I. (2017). EEG brain activity in dynamic health qigong training: same effects for mental practice and physical training? Front. Psychol. 8:154. doi: 10.3389/fpsyg.2017.00154, PMID: 28223957 PMC5293832

[ref40] Herzog H. Lele V. R. Kuwert T. Langen K. J. Rota Kops E. Feinendegen L. E. (1990-1991). Changed pattern of regional glucose metabolism during yoga meditative relaxation. Int. Pharmacopsychiatry 23, 182–187. doi: 10.1159/000119450, PMID: 2130287

[ref41] Holzel B. K. Ott U. Gard T. Hempel H. Weygandt M. Morgen K.. (2008). Investigation of mindfulness meditation practitioners with voxel-based morphometry. Soc. Cogn. Affect. Neurosci. 3, 55–61. doi: 10.1093/scan/nsm038, PMID: 19015095 PMC2569815

[ref42] Holzel B. K. Ott U. Hempel H. Hackl A. Wolf K. Stark R.. (2007). Differential engagement of anterior cingulate and adjacent medial frontal cortex in adept meditators and non-meditators. Neurosci. Lett. 421, 16–21. doi: 10.1016/j.neulet.2007.04.074, PMID: 17548160

[ref43] Josipovic Z. (2010). Duality and non-duality in meditation research. Conscious. Cogn. 19, 1119–1121. doi: 10.1016/j.concog.2010.03.016, PMID: 20385506

[ref44] Josipovic Z. (2013). Neural correlates of nondual awareness in meditation. Ann. N. Y. Acad. Sci. 1307, 9–18. doi: 10.1111/nyas.12261, PMID: 24033505

[ref45] Josipovic Z. (2019). Nondual awareness: consciousness-as-such as non-representational reflexivity. Prog. Brain Res. 244, 273–298. doi: 10.1016/bs.pbr.2018.10.021, PMID: 30732841

[ref46] Josipovic Z. Dinstein I. Weber J. Heeger D. J. (2011). Influence of meditation on anticorrelated networks in the brain. Front. Hum. Neurosci. 5:183. doi: 10.3389/fnhum.2011.00183, PMID: 22287947 PMC3250078

[ref47] Josipovic Z. Miskovic V. (2020). Nondual awareness and minimal phenomenal experience. Front. Psychol. 11:2087. doi: 10.3389/fpsyg.2020.02087, PMID: 32973628 PMC7473343

[ref48] Kendig C. Witteveen J. (2020). The history and philosophy of taxonomy as an information science. Hist Philos Life Sci 42:40. doi: 10.1007/s40656-020-00337-8 32865722

[ref49] Khalsa D. H. Amen D. Hanks C. Money N. Newberg A. (2009). Cerebral blood flow changes during chanting meditation. Nucl. Med. Commun. 30, 956–961. doi: 10.1097/MNM.0b013e32832fa26c, PMID: 19773673

[ref50] Koshikawa F. Ichii M. Ishii Y. Suzuki M. (1996). “An experiment on classifications of meditation methods” in Comparitive and Psychological Study on Meditation. eds. Haruki Y.. (Delft: Eburon Publishers), 213–223.

[ref51] Kumar U. Guleria A. Kishan S. S. K. Khetrapal C. L. (2014). Effect of SOHAM meditation on human brain: a voxel-based morphometry study. J. Neuroimaging 24, 187–190. doi: 10.1111/jon.12040 23773541

[ref52] Lazar S. W. Bush G. Gollub R. L. (2000). Functional brain mapping of the relaxation response and meditation. Neuroreport 11, 1581–1585. doi: 10.1097/00001756-200005150-00042, PMID: 10841380

[ref53] Lee T. M. C. Leung M.-K. Hou W.-K. Tang J. C. Y. Yin J. So K.-F.. (2012). Distinct neural activity associated with focused-attention meditation and loving-kindness meditation. PLoS One 7:e40054. doi: 10.1371/journal.pone.0040054, PMID: 22905090 PMC3419705

[ref54] Lehmann D. Faber P. L. Achermann P. Jeanmonod D. Gainotti L. Pizzagalli D. (2001). Brain sources of EEG gamma frequency during volitionally meditation-induced, altered states of consciousness, and experience of the self. Psychiatry Res. Neuroimaging 108, 111–121. doi: 10.1016/S0925-4927(01)00116-0, PMID: 11738545

[ref55] Leung M.-K. Chan C. Yin J. Lee C.-F. So K.-F. Lee T. (2013). Increased gray matter volume in the right angular and posterior parahippocampal gyri in loving-kindness meditators. Soc. Cogn. Affect. Neurosci. 8, 34–39. doi: 10.1093/scan/nss076, PMID: 22814662 PMC3541494

[ref56] Lou H. Kjaer T. W. Friberg L. Wildschiodtz G. Holm S. Nowa M. (1999). A 15O-H2O PET study of meditation and the resting state of normal consciousness. Hum. Brain Mapp. 7, 98–105. doi: 10.1002/(SICI)1097-0193(1999)7:2<98::AID-HBM3>3.0.CO;2-M, PMID: 9950067 PMC6873339

[ref57] Lubischew A. (1969). Philosophical aspects of taxonomy. Annu. Rev. Entomol. 14, 19–38. doi: 10.1146/annurev.en.14.010169.000315

[ref58] Lutz A. Brefczynski-Lewis J. Johnstone T. Davidson R. J. (2008). Regulation of the neural circuitry of emotion by compassion meditation: effects of meditative expertise. PLoS One 3:e1897. doi: 10.1371/journal.pone.0001897, PMID: 18365029 PMC2267490

[ref59] Lutz A. Dunne J. D. Davidson R. J. (2007). “Meditation and the neuroscience of consciousness” in The Cambridge Handbook of Consciousness. eds. Zelazo P. D. Moscovitch M. Thompson E. (Cambridge: Cambridge University Press), 499–552.

[ref60] Lutz A. Greischar L. Rawlings N. Ricard M. Davidson R. (2004). Long-term meditators self-induce high-amplitude gamma synchrony during mental practice. Proc. Natl. Acad. Sci. U. S. A. 101, 16369–16373. doi: 10.1073/pnas.0407401101, PMID: 15534199 PMC526201

[ref61] Lutz A. Jha A. P. Dunne J. D. Saron C. D. (2015). Investigating the phenomenological matrix of mindfulness related practices from a neurocognitive perspective. Am. Psychol. 70, 632–658. doi: 10.1037/a0039585, PMID: 26436313 PMC4608430

[ref62] Maharishi Mahesh Yogi. (1986). Life Supported by Natural Law. Wash. D.C.: Age of Enlightenment Press

[ref63] Mascaro J. S. Rilling J. K. Tenzin Negi L. Raison C. L. (2013). Compassion meditation enhances empathic accuracy and related neural activity. Soc. Cogn. Affect. Neurosci. 8, 48–55. doi: 10.1093/scan/nss095, PMID: 22956676 PMC3541495

[ref64] Matko K Ott U., and Sedlmeier P. (2018). The top 10: prevalence and popularity of basic meditation practices in different spiritual traditions. International Conference of Mindfulness, Amsterdam.

[ref65] Matko K. Ott U. Sedlmeier P. (2021). What do meditators do when they meditate? Proposing a novel basis for future meditation research. Mindfulness 12, 1791–1811. doi: 10.1007/s12671-021-01641-5

[ref66] Matko K. Sedlmeier P. (2019). What is meditation? Proposing an empirically derived classification system. Front. Psychol. 10:2276. doi: 10.3389/fpsyg.2019.02276, PMID: 31681085 PMC6803504

[ref67] Mayr E. (1969). Principles of Systematic Zoology. Cambridge, MA: Harvard Univ. Press

[ref69] Moss A. S. Wintering N. Roggenkamp H. Khalsa D. S. Waldman M. R. Monti D.. (2012). Effects of an 8-week meditation program on mood and anxiety in patients with memory loss. J. Integrat. Complement. Med. 18, 48–53. doi: 10.1089/acm.2011.0051, PMID: 22268968

[ref70] Muktananda S. (1978) in I am That. ed. Fallsburg S. (N.Y: SYDA Publishers)

[ref71] Muktananda S. (1980). Meditate. Albany N.Y: SUNY Press

[ref72] Muncy J. A. (1986). Affect and cognition: a closer look at two competing theories. Adv. Consum. Res. 13, 226–230.

[ref73] Nash J. Newberg A. Awasthi B. (2013). Toward a universal definition and taxonomy for meditation. Front. Psychol. 4:806. doi: 10.3389/fpsyg.2013.00806, PMID: 24312060 PMC3834522

[ref74] Neander K. Schulte P. (2020). “Teleological theories of mental content” in Stanford Encyclopedia of Philosophy. ed. Zalta E. N.

[ref75] Newberg A. d’Aquili E. (2001). Why God Won’t Go Away. New York, NY: Ballantine Publishing Group

[ref76] Newberg A. Iversen J. (2003). The neural basis of the complex mental task of meditation: neurotransmitter and neurochemical considerations. Med. Hypotheses 61, 282–291. doi: 10.1016/S0306-9877(03)00175-0, PMID: 12888320

[ref77] Newberg A. Pourdehnad M. Alavi A. d’Aquili E. (2003). Cerebral blood flow during meditative prayer: preliminary findings and methodological issues. Percept. Mot. Skills 97, 625–630. doi: 10.2466/pms.2003.97.2.625, PMID: 14620252

[ref78] Newberg A. Wintering N. Khalsa D. S. Roggenkamp H. Waldman M. R. (2010). Meditation effects on cognitive function and cerebral blood flow in subjects with memory loss: a preliminary study. J. Alzheimers Dis. 20, 517–526. doi: 10.3233/JAD-2010-1391, PMID: 20164557

[ref79] Osho (2003). The ABC of Enlightenment. London: Harper Collins

[ref80] Ospina M. B. Bond K. Karkhaneh M. Tjosvold L. Vandermeer B. Liang Y.. (2007). Meditation practices for health: state of the research. Evidence reports/technology assessments. No. 155, Rockville, MD: Agency for Healthcare Research and Quality. PMC4780968 17764203

[ref81] Panda R. Bharath R. D. Upadhyay N. Mangalore S. Chennu S. Rao S. L. (2016). Temporal dynamics of the default mode network characterize meditation-induced alterations in consciousness. Front. Hum. Neurosci. 10:372. doi: 10.3389/fnhum.2016.00372, PMID: 27499738 PMC4956663

[ref82] Paoletti P. Ben-Soussan T. D. (2020). Reflections on inner and outer silence and consciousness without contents according to the sphere model of consciousness. Front. Psychol. 11:1807. doi: 10.3389/fpsyg.2020.01807, PMID: 32903475 PMC7435012

[ref83] Peres J. F. Giommi F. Gielen S. C. (2012). Neuroimaging during trance state: a contribution to the study of dissociation. PLoS One 7:e49360. doi: 10.1371/journal.pone.0049360, PMID: 23166648 PMC3500298

[ref84] Pilla D. Qina’au J. Patel A. Meddaoui B. Watson N. Dugad S.. (2020). Toward a framework for reporting and differentiating key features of meditation-and mindfulness-based interventions. Mindfulness 11, 2613–2628. doi: 10.1007/s12671-020-01475-7

[ref85] Powell R. (ed.). (1994). The Ultimate Medicine—As Prescribed by Sri Nisargadatta Maharaj. San Diego: Blue Dove Press

[ref86] Powell R. (1996). The Experience of Nothingness: Sri. Nisargadatta Maharaj’s Talks on Realizing the Infinite. San Diego: Blue Dove Press

[ref87] Prabhavananda S Manchester F. (1948). The Upanishads: Breath of the Eternal. New York: New Amer. Library

[ref88] Raffone A. Marzetti L. Del Gratta C. Perruccib M. G. Romani G. L. Pizzella V. (2019). Toward a brain theory of meditation. Prog. Brain Res. 244, 207–232. doi: 10.1016/bs.pbr.2018.10.028 30732838

[ref89] Reitan R. Wolfson D. (2000). Conation: a neglected aspect of neuropsychological functioning. Arch. Clin. Neuropsychol. 15, 443–453. doi: 10.1016/S0887-6177(99)00043-8, PMID: 14590220

[ref90] Saggar M. King B. G. Zanesco A. P. (2012). Intensive training induces longitudinal changes in meditation state-related EEG oscillatory activity. Front. Hum. Neurosci. 6:256. doi: 10.3389/fnhum.2012.00256, PMID: 22973218 PMC3437523

[ref91] Schwitzgebel E. (2010). “Introspection” in The Stanford Encyclopedia of Philosophy. ed. Zalta E.

[ref92] Shankarananda S. (2003). Consciousness is Everything: The Yoga of Kashmir Shaivism. Melbourne, VIC: Shaktipat Press

[ref93] Sokal R. Sneath P. (1963). The Principles of Numerical Taxonomy. San Francisco: W. H. Freeman Publishers

[ref94] Sparby T. Sacchet M. (2022). Defining meditation: foundations for an activity-based phenomenological classification system. Front. Psychol. 12:795077. doi: 10.3389/fpsyg.2021.795077, PMID: 35153920 PMC8832115

[ref95] Srinivasan N. (2020). Consciousness without content: a look at evidence and prospects. Front. Psychol. 11:1992. doi: 10.3389/fpsyg.2020.01992, PMID: 32849160 PMC7426455

[ref96] Thibault R. Lifshitz M. Raz A. (2014). Posture alters human resting-state. Cortex 58, 199–205. doi: 10.1016/j.cortex.2014.06.014 25041937

[ref35] Thich Nhat Hahn. (1991). Peace is Every Step. ed. A. Kotler (New York, N.Y: Bantam Books)

[ref97] Tomasino B. Chiesa A. Fabbro F. (2014). Disentangling the neural mechanisms involved in Hinduism-and Buddhism-related meditations. Brain Cogn. 90, 32–40. doi: 10.1016/j.bandc.2014.03.013, PMID: 24975229

[ref98] Travis F. (2020). On the neurobiology of meditation: comparison of three organizing strategies to investigate brain patterns during meditation practice. Medicina 56:712. doi: 10.3390/medicina56120712, PMID: 33353049 PMC7767117

[ref100] Travis F. Nash J. Parim N. Cohen B. H. (2020). Does the MRI/fMRI procedure itself confound the results of meditation research? Front. Psychol. 11:728. doi: 10.3389/fpsyg.2020.00728, PMID: 32411046 PMC7198852

[ref101] Travis F. Pearson C. (2000). Pure consciousness: distinct phenomenological and physiological correlates of “consciousness itself.” Int. J. Neurosci. 100, 77–89. doi: 10.3109/00207450008999678 10512549

[ref102] Travis F. Shear J. (2010). Focused attention, open monitoring and automatic self-transcending: categories to organize meditations from Vedic, Buddhist and Chinese traditions. Conscious. Cogn. 19, 1110–1118. doi: 10.1016/j.concog.2010.01.007, PMID: 20167507

[ref103] Vaitl D. Birbaumer N. Gruselier J. Jamieson G. Lehmann D. Ott U.. (2005). Psychobiology of altered states of consciousness. Psychol. Bull. 131, 98–127. doi: 10.1037/0033-2909.131.1.98, PMID: 15631555

[ref104] Walsh D. (2006). Evolutionary essentialism. Br. J. Philos. Sci. 57, 425–448. doi: 10.1093/bjps/axl001

[ref105] Wang D. Rao H. Korczykowski M. Wintering N. Pluta J. Khalsa D. S.. (2011). Cerebral blood flow changes associated with different meditation practices and perceived depth of meditation. Psychiatry Res. Neuroimaging 191, 60–67. doi: 10.1016/j.pscychresns.2010.09.011, PMID: 21145215

[ref106] West M. A. (1987). “Traditional and psychological perspectives on meditation” in The Psychology of Meditation. ed. West M. A. (Oxford: Clarendon Press), 5–22.

[ref107] Winter U. LeVan P. Borghardt T. Akin B. Wittmann M. Leyens Y.. (2020). Content-free awareness: EEG-fcMRI correlates of consciousness as such in an expert meditator. Front. Psychol. 10:3064. doi: 10.3389/fpsyg.2019.03064, PMID: 32132942 PMC7040185

[ref108] Woods T. J. Windt J. Carter O. (2022). Evidence synthesis indicates contentless experiences in meditation are neither truly contentless nor identical. Phenomenol. Cogn. Sci. 1-52. doi: 10.1007/s11097-022-09811-z

[ref109] Yordanova J. Kolev V. Mauro F. Nicolardi V. Simione L. Calabrese L.. (2020). Common and distinct lateralized patterns of neural coupling during focused attention, open monitoring and loving kindness meditation. Sci. Rep. 10:7430. doi: 10.1038/s41598-020-64324-6, PMID: 32366919 PMC7198563

[ref110] Yordanova J. Kolev V. Nicolardi V. Simione L. Mauro F. Garberi P.. (2021). Attentional and cognitive monitoring brain networks in long-term meditators depend on meditation states and expertise. Sci. Rep. 11:4909. doi: 10.1038/s41598-021-84325-3, PMID: 33649378 PMC7921394

[ref111] Zaccaro A. Riehl A. Piarulli A. Alfì G. Neri B. Menicucci D.. (2021). The consciousness state of traditional Nidra yoga/modern yoga Nidra: phenomenological characterization and preliminary insights from an EEG study. Int. J. Yoga. Therap. 31:14. doi: 10.17761/2021-D-20-00014, PMID: 34727178

